# Pharmacokinetic Modeling to Guide Preclinical Development of an Islatravir-Eluting Reservoir-Style Biodegradable Implant for Long-Acting HIV PrEP

**DOI:** 10.3390/pharmaceutics16020201

**Published:** 2024-01-30

**Authors:** Talisa S. Kinsale, Mackenzie L. Cottrell, Linying Li, Rhonda Brand, Greg Gatto, Ellen Luecke, Chasity Norton, Archana Krovi, Julie B. Dumond, Gauri Rao, Shekhar Yeshwante, Brian Van Horne, Ariane Van Der Straten, Angela D. M. Kashuba, Leah M. Johnson

**Affiliations:** 1 Division of Pharmacotherapy and Experimental Therapeutics, Eshelman School of Pharmacy, University of North Carolina, Chapel Hill, NC 27599, USA; tkinsale@email.unc.edu (T.S.K.); jdumond@unc.edu (J.B.D.); gaurirao@live.unc.edu (G.R.); shekhary@email.unc.edu (S.Y.); bvanhorn@email.unc.edu (B.V.H.); akashuba@unc.edu (A.D.M.K.); 2 Biomedical Technologies RTI International, 3040 E. Cornwallis Road, Research Triangle Park, NC 27709, USA; ali@rti.org (L.L.); cnorton@rti.org (C.N.); akrovi@rti.org (A.K.);; 3 Magee-Womens Research Institute, University of Pittsburgh, Pittsburgh, PA 15213, USA; rmb91@pitt.edu; 4 Global Public Health Impact Center, RTI International, Research Triangle Park, NC 27709, USA; ggatto@rti.org (G.G.); eluecke@rti.org (E.L.); 5 Center for AIDS Prevention Studies (CAPS), Department of Medicine, University of California San Francisco, San Francisco, CA 94104, USA; ariane.vanderstraten@ucsf.edu; 6 ASTRA Consulting, Kensington, CA 94708, USA

**Keywords:** HIV prevention, preexposure prophylaxis, antiretroviral agents, islatravir, extended-duration, long-acting implant

## Abstract

Long-acting injectable cabotegravir is more effective than daily oral PrEP at preventing HIV transmission due to improved adherence, but requires bi-monthly large-volume intramuscular injections. Subcutaneous (SC) contraceptive implants can be formulated with antiretrovirals for extended-duration HIV PrEP. Islatravir (ISL) is a first-in-class, investigational antiretroviral with pharmacologic properties well-suited for implant delivery. We performed preclinical studies for the development of a reservoir-style, poly(ε-caprolactone) ISL-eluting implant by conducting a single-dose SC ISL dose-ranging pharmacokinetic (PK) study of 0.1, 0.3, and 1 mg/kg in adult Wistar rats. Non-compartmental analysis was conducted, and dose proportionality assessed for ISL plasma and intracellular islatravir-triphosphate (ISL-tp). Population PK models estimated ISL’s unit impulse response to deconvolve ISL-implant in vivo absorption rate (mg/day) and cumulative mass (mg) from published rat plasma PK (*n* = 10). Drug release was interpreted using four kinetic models. Dose proportionality was affirmed for ISL and ISL-tp. A first-order, two-compartment model fitted the SC ISL bolus data. Mean (SD) absorption rate from 0 to 154 days was 0.072 ± 0.024 mg/day, and cumulative mass at 154 days was 8.67 ± 3.22 mg. ISL absorption was well-described by zero-order (r^2^ = 0.95) and Ritger–Peppas (r^2^ = 0.98). Our zero-order ISL-release poly(ε-caprolactone) implant is projected to achieve clinical PK above ISL-tp’s PrEP efficacy threshold. Continued development for HIV PrEP applications is warranted.

## 1. Introduction

Fixed-dose combinations of daily oral antiretrovirals (emtricitabine 200 mg with tenofovir disoproxil fumarate 300 mg or tenofovir alafenamide 25 mg) have been shown to reduce the risk of HIV acquisition in multiple randomized placebo-controlled trials (RCT) and their open label extensions [1,2]. However, two large oral preexposure prophylaxis (PrEP) trials enrolling exclusively cisgender female populations were stopped early due to futility—later attributed to incomplete adherence to the study treatment [3,4]. Recently, a long-acting (LA) injectable formulation of the ARV, cabotegravir, demonstrated superiority over oral PrEP in preventing HIV acquisition in both male and female populations [5,6]. While injectable LA PrEP is more effective than oral PrEP due to improved adherence, it requires a large-volume (3 mL) intramuscular injection every other month with injection site reactions reported by 50% of patients [7]. Additionally, this injectable exhibits a sustained depletion phase (termed the PK tail), where the ARV (e.g., cabotegravir) is detectable at subtherapeutic levels [8]. This tail increases the risk of breakthrough infection with ARV resistance in patients who choose to discontinue LA PrEP or fall out of care.

Subcutaneous implants, such as those used for hormonal contraceptives, can offer therapeutic advantages over currently approved drug delivery approaches for LA PrEP [9]. Advantages include the potential for extended therapeutic intervals, retrievability in the event of adverse drug reactions or alternative reasons to discontinue PrEP, and improved adherence. The design of an implant comprising biodegradable polymers, such as poly(ε-caprolactone) (PCL), offers an additional advantage of bypassing the need for device removal, which could prove desirable for health care providers and end-users in HIV PrEP applications [10,11].

Islatravir (4′-ethynyl-2-fluoro-2′-deoxyadenosine [ISL]) is a prodrug, first-in-class nucleoside reverse transcriptase translocation inhibitor (NRTTI) deoxyadenosine analog that suppresses wild type HIV-1 virus at subnanomolar concentrations [12]. Its activity in preclinical and clinical studies demonstrated its antiviral potency as an investigational PrEP agent [13], and the active islatravir triphosphate (ISL-tp) metabolite exhibited an extended intracellular half-life (120–177 h) following oral dosing [14,15]. Pharmacokinetic/pharmacodynamic (PK/PD) modeling of clinical and preclinical data estimated an inhibitory quotient (IQ = 5) for ISL-tp, which defined a therapeutic threshold of 0.05 pmol/million peripheral blood mononuclear cells (PBMCs) for prevention of HIV-1 acquisition [16].

PrEP trials of an ISL-eluting prototype polymer implant (54 mg and 62 mg) [17] and downstream next-generation radiopaque [18] implant maintained ISL-tp PBMC concentrations above the PrEP efficacy threshold for 12 weeks. Secondary safety endpoints were similar to those observed in earlier oral studies, with no discontinuations due to adverse events [19]. In 2021, clinical trials evaluating ISL were placed on hold following findings of decreased CD4^+^ T cell and total lymphocyte counts in participants taking oral ISL that ranged from 0.75 mg daily to 120 mg monthly [20]. Subsequent meta-analyses and modeling demonstrated a dose-dependent relationship for this adverse event and established 0.25 mg daily as a safe upper dosing limit for the oral formulation [21]. Importantly, clinical trials evaluating the ISL-eluting implants did not report decreased CD4^+^ T cells likely due to the tested release rates in these studies falling below this upper dosing limit [17,18].

A biodegradable reservoir-style implant comprised of PCL has been previously formulated to co-deliver contraceptive hormone and ISL for 6 months in rats [22]. Dose optimization efforts for this type of controlled-release formulation can be enhanced through characterization of the mass transport mechanisms dictating drug dissolution. These mechanisms have been previously described by well-defined empirical mathematical equations such as zero-order, first-order, Higuchi and Ritger–Peppas.

Here, we conducted a preclinical SC bolus study in rats for deconvolution of ISL’s in vivo absorption PK to optimize implant release rates for LA PrEP. We leveraged PK modeling to fully characterize the in vivo absorption and distribution kinetics as a guide to interspecies dose-translation efforts during development of a long-acting controlled-release implant for HIV PrEP.

## 2. Materials and Methods

### 2.1. Drug Preparation

ISL was provided by Pharmaron (Beijing, China). Drug purity was determined on a 5-point calibration curve by linear regression analysis to be 97.3% and titrated as 1 mg/mL stock solutions in DMSO at 37 °C and prepared 24 h prior to administration.

### 2.2. Preclinical Islatravir PK Study 

A SC bolus animal study was conducted to match the species and route of administration for the published SC ISL-eluting implant study under the University of Pittsburgh Institutional Animal Care and Use Committee (IACUC) (Protocol 20056840) at Magee-Womens Research Institute and Foundation (Pittsburgh, PA, USA), in collaboration with Biomedical Technologies (RTI International, Research Triangle Park, NC, USA). All procedures met the principles of the NIH Office of Laboratory Animal Welfare, Guide for the Care and Use of Laboratory Animals [23]. Seventy-two adult female Wistar rats with mean (±standard deviation, SD) body weight of 0.307 ± 0.02 kg, were included in this dose-ranging study designed to characterize the 24 h plasma and PBMC PK profile for three ISL doses, 0.1 mg/kg (low), 0.3 mg/kg (mid), and 1 mg/kg (high), selected to encompass the projected daily dose from the published optimized implant design [22].

### 2.3. Dose Administration and Sparse Sampling Schema

Animals were observed for general health daily and weighed prior to each dose. Sparse timepoints collected were 0.17, 0.5, 1, 2, 4, 6, and 24 h for each dose level. Predose samples were collected before ISL bolus administration, and all were below limits of quantification. For a single timepoint, three rats were administered a SC bolus under the neck skin and sampled at each pre-determined timepoint, contributing one measure per animal. The dosing schedule within each dose level was staggered to reduce blood loss so that each subset (*n* = 3) of animals contributed a single measure per timepoint within the 24 h period. Blood was drawn from the lateral tail vein at each set timepoint (1.2–1.7 mL), and samples were processed to plasma or PBMCs by centrifugation for the determination of ISL and ISL-tp using previously published procedures [22].

### 2.4. Islatravir Quantification

ISL was quantified in plasma and ISL-tp in PBMC cell lysate using previously published liquid-chromatography tandem mass spectrometry (LC-MS/MS) methods [22,24]. In short, ISL and ISL-tp were prepared in 100 μL of 70:30 methanol/water and extracted with isotopically labeled internal standards (IS-^3^C^15^N_3_-ISL and ^3^C_10_,^15^N_5_-dGTP, respectively) and detected on an AB Sciex API-5000 triple quadrupole mass spectrometer under positive ionization mode (SCIEX, Framingham, MA, USA). A dynamic calibration range of 0.1–100 ng/mL was used for ISL with precision and accuracy acceptance criteria within ±15%. ISL-tp was analyzed using the same mass spectrometer calibrated for 0.05–125 ng/mL (ISL-tp). ISL-tp concentrations were normalized to representative live cell counts determined by Trypan blue staining and a hemocytometer and reported as pmol/million cells. Concentrations below the limit of quantification (LLOQ) for each were censored according to the Beal M2 method [25].

### 2.5. Noncompartmental Analysis

SC bolus plasma ISL (ng/mL) and intracellular ISL-tp (pmol/million cells) concentration data were visually inspected on a concentration–time plot. A sparse, extravascular, weight (kg) normalized, non-compartmental analysis (NCA) was used to estimate pharmacokinetic endpoints for maximal concentration (C_max_), time to C_max_ (T_max_) and area under the curve to the last measurable timepoint (AUC_last_) by linear up-log down trapezoidal method. Half-life (t_1/2_) was estimated from 4 to 24 h for both ISL and ISL-tp profiles that had 3 quantifiable datapoints, except the low dose (0.1 mg/kg) plasma ISL. The average concentration (C_avg_) of plasma ISL and PBMC ISL-tp were estimated by NCA estimated AUC divided by 24 h. The ratio of ISL-tp:ISL was calculated assuming an approximate PBMC volume of 0.2 pL per cell determined for 1 million live cells in each PBMC sample [17]. Dose proportionality was assessed for ISL and ISL-tp by unweighted linear regression of the natural log (ln) transformed parameters, C_max_ and AUC_last_, versus ln transformed absolute dose (mg) of each dose level. The slope was reported, and linearity was declared for values approximating 1 and coefficient of r^2^ > 0.8.

### 2.6. Pharmacokinetic Analysis

We tested 1- and 2-compartment, macro parameterized population PK (PopPK) models with first-order linear kinetics fit to the SC bolus plasma ISL concentration data for individual dose levels (0.1, 0.3, 1 mg/kg) and a collapsed simultaneous model of all dose levels. Each population model was conducted with a naive pooled algorithm for multiplicative, additive, and log-additive error models. They were each evaluated by: (1) goodness-of-fit plots (residual, predicted and quantile-quantile), (2) model diagnostics (log-likelihood and akaike/Bayesian criterion), and (3) reduction in parameter/model variability (%CV < 30) for the final model. To enhance robustness, each model eta (η) diagnostics were tested for shrinkage (>0.3) and nested models were additionally prioritized by minimum objective function value. The final popPK model estimates for macro-constants (A, B ng/mL) were divided by the average dose (145,803 ng) to attain the unit impulse response (UIR) for plasma ISL in rats. Each dose profile was assessed for outliers by Grubb’s test which were excluded, and PK analyses were performed in Phoenix NLME^TM^ v6.3 (Certara, Inc., Princeton, NJ, USA), with estimates reported as mean ± SD per dose level.

### 2.7. Deconvolution of In Vivo Absorption

To perform deconvolution, we utilized previously published plasma PK profiles from 10 adult Wistar rats with a SC ISL-eluting implant (40 mm long, 100 μm wall-thickness) comprised of extruded PCL from PURASORB PC-17 pellets (106 kDa) (Amsterdam, The Netherlands) formulated with a 1:1 mass ratio of ISL: sesame oil [22]. In this previously published study animals were treated with either ISL-only, which included one ISL-eluting implant and one excipient-only implant; or ISL-plus with one ISL-eluting implant and one etonogestrel (ENG)-eluting implant (hormone data not discussed here). While PK samples were collected over 197 days of implantation in this previous published study, increasing inter-individual variability (%CV > 90) in plasma concentrations was observed beyond 100 days, which may have been associated with decreased mechanical integrity of the implants. As such, the deconvolution analyses for in vivo drug absorption were constrained to 154 days (%CV ≤ 60). Two animals were identified as outliers contributing >30% variability to the mean PK profiles and were excluded from downstream analyses. The 2-compartmental model-derived UIR was used to deconvolve the individual in vivo animal absorption profiles to estimate: (1) rate of absorption (mg/day), (2) cumulative mass absorbed (mg), and (3) fraction of mass released from the implant loaded dose. Welch’s *t*-test was utilized to compare the rate of absorption for ISL-only and ISL-plus groups at 90 and 154 days. Drug recovery was calculated as mean cumulative mass absorbed (mg) at day 154 subtracted from mean loaded dose (98.6 ± 3.31 mg) in the reservoir-style PCL implant. Analyses were performed in Phoenix WinNonlin^TM^ Deconvolution Toolkit v6.3 (Certara, Inc., Princeton, NJ, USA) and reported as mean ± SD for each profile.

### 2.8. In Vivo Drug Absorption Models

Plasma ISL in vivo absorption kinetics were characterized by fitting the individual animal profiles (*n* = 10) of deconvolved cumulative mass (mg) absorbed over time (days) to 4 kinetic models describing drug distribution from the published ISL-eluting PCL implant [22]. Cumulative mass (mg) data were fit to (pseudo)linear equations of each kinetic model using SAS^®^ Studio v9.04 (Enterprise 3.81, SAS Institute, Cary, NC, USA) to fit regression models to their release profiles using the “proc model” function (Figure S1). The individual r-squared (r^2^) correlation coefficient was determined for each animal profile (*n* = 10) and prioritized by highest mean r^2^ with a minimum threshold of r^2^ > 0.75. For the Ritger–Peppas model the drug release exponent, *n*, was characterized to describe mechanisms of non-Fickian release. The mathematical equations describing each model for: Zero-order (constant release); First-order (concentration-dependent release with respect to time); Higuchi (square root of a time-dependent release from planar matrix systems); and Ritger–Peppas (power law exponential diffusion from polymeric systems) were:

*M_t_* = *M*_0_ + *K_Z_t*, Zero-order (1)

*M_t_* = *M*_0_ + *K_F_t*, First-order (2)

*M_t_* = *M*_0_ + *K_H_t*^0.5^, Higuchi (3)

*M_t_*/*M_inf_* = *K_R_t^n^*, Ritger–Peppas (4)

where *M_t_* is the cumulative mass absorbed from the implant at time *t, M*_0_ is the mass of drug absorbed at *t* = 0 (attributable to instantaneous burst release)*, M_inf_* is the total dug loaded in the implant, *n* is the power exponential (Ritger–Peppas only), and *K* is the absorption rate constant for each respective model estimated by regression analysis. We assumed that *M*_0_ was negligible for this reservoir-style implant system. Each mathematical model was leveraged as reported by Bruschi et al. and Costa et al., to characterize in vivo drug absorption kinetics of ISL from the PCL implant [26,27]. 

## 3. Results

### 3.1. Noncompartmental PK Analysis

**Figure 1:**
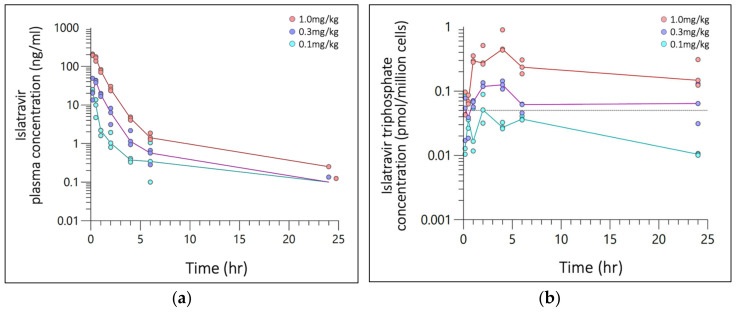
Semi-log concentration–time plot of (**a**) Islatravir (ISL) plasma concentration (ng/mL) and (**b**) ISL-triphosphate (ISL-tp) concentration (pmol/million cells) following a single SC administration at 0.1, 0.3, or 1.0 mg/kg. Plasma and PBMCs was collected from 3 rats per time point, with 24 rats per dosing level. Markers are individual plasma concentration with median (line) categorized by dose (color). The grey reference line (dash) is clinical threshold of 0.05 pmol/million cells.

SC bolus plasma concentrations were plotted against time for *n* = 57 quantifiable ISL values. Visual inspection of each concentration–time profile elucidated a multi-phasic drug distribution and clearance (Figure 1a); 0–5 h for the first phase, and 5 h onwards for the second, excluding the lowest dose level which was not quantifiable at 24 h. Six values were below the assay limit of quantification (BLQ), and one outlier (confirmed by Grubb’s test) was censored. The mean T_max_ was at 10 min except for the mid dose level (0.3 mg/kg) which occurred at 30 min. Estimates for C_max_, C_avg_ and AUC_last_ were: 22.6, 44.7, 201.3 ng/mL, 0.675; 2.283, 9.566 ng/mL; and 16.2, 54.8, 229.6 h*ng/mL for dose levels 0.1, 0.3, 1.0 mg/kg, respectively. Plasma ISL C_max_ and AUC_last_ exhibited dose proportionality with slopes of 0.95 (r^2^ = 0.961) and 1.15 (r^2^ = 0.995) for ln[Dose] vs. ln[C_max_] and ln[AUC_last_], respectively.

SC bolus ISL-tp PBMC concentrations were plotted against time for *n* = 43 quantifiable ISL-tp values (Figure 1b) and 20 BLQ values. Time to maximal concentration ranged from 2–4 h and t_1/2_ was 11.8, 27.1, 17.8 hr^−1^, respectively. Estimates for C_max_, C_avg_ and AUC_last_ were: 0.057, 0.125, 0.593 pmol/million cells; 0.025, 0.073, 0.254 pmol/million cells; and 0.609, 1.746, 6.086 h*pmol/million cells for dose levels 0.1, 0.3, 1.0 mg/kg, respectively. PBMC ISL-tp C_max_ and AUC_last_ exhibited dose proportionality with slopes of 1.02 (r^2^ = 0.974) and 0.99 (r^2^ = 0.998) for ln[Dose] vs. ln[Cmax] and ln[AUC_last_], respectively. The ratio of C_avg_ for ISL-tp:ISL for 0.1, 0.3 and 1.0 mg/kg doses were 100, 85 and 70, respectively.

### 3.2. Pharmacokinetic Modeling

**Figure 2:**
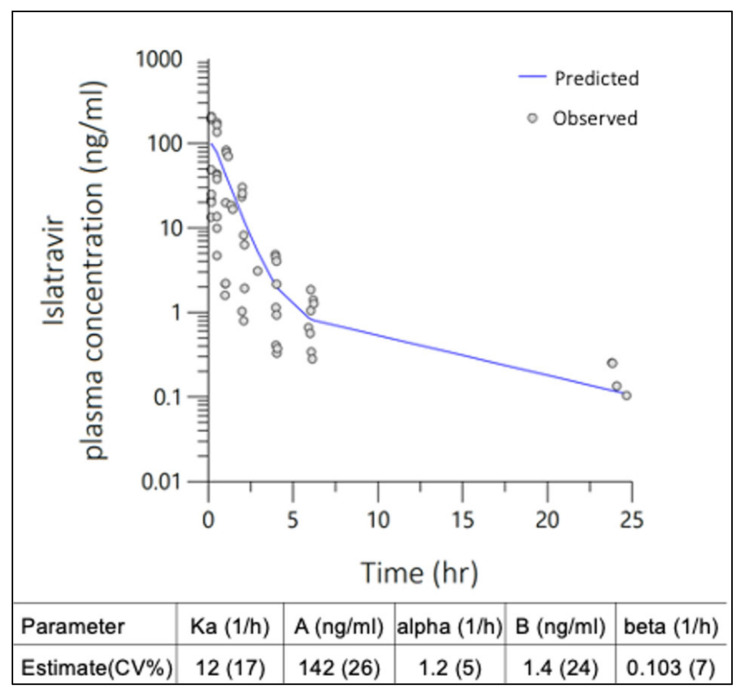
Two-compartment population pharmacokinetic (PopPK) model with multiplicative error was fit to ISL plasma concentrations observed in rats following single SC administration at 0.1, 0.3 and 1 mg/kg doses. Macro-parameter constants (A, B) and exponential rates (α, β) are described in the inset table.

The 24 h plasma-concentration PK profile for all SC ISL bolus doses were best described by a simultaneously modeled two-compartment, multiplicative error, naïve pooled PopPK model, with 76% residual error (Figure 2) for all dose levels. Extravascular absorption rate (Ka) was >10 h^−1^ for individual dose level models and estimated at 12 h^−1^ for the final simultaneously modeled extravascular two-comp PopPK macro-model. The popPK macro-parameters were fit to each compartment describing the parameter estimates for determining the UIR of ISL in rats after subcutaneous administration (Figure 2, inset table).

**Figure 3:**
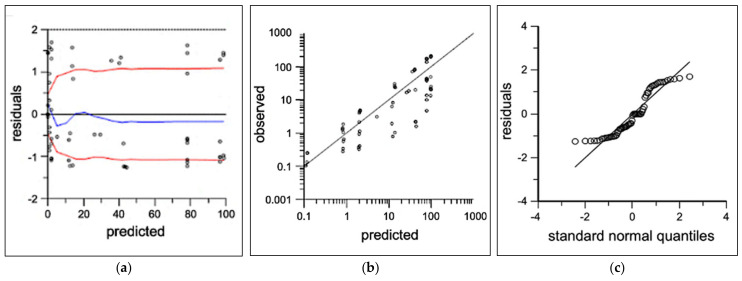
Two-compartment population PK diagnostics showing observed (open circles) and predicted (black lines) for (**a**) individual predicted concentrations vs. residuals with loess fit (blue) and absolute trend lines (red), (**b**) individual predicted vs. observed log-concentrations, and (**c**) quantile–quantile normal plot.

No preclusive deviations from normality were identified in the final model diagnostic plots which were evaluated for; (Figure 3a) individual unweighted residual distribution, (Figure 3b) observed–predicted unweighted distribution about the line of unity, and (Figure 3c) the quantile–quantile plot describing the normality of the model residuals. Lower concentrations in the 0.1 mg/kg dose level approaching the limit of quantification had overall negligible bias in the final model. Tailing of the quantiles indicated some deviations from normality in the extremities. Overall, the diagnostics show adequate fit for fit-for-purpose deconvolution, with best prediction for doses 0.3 mg/kg and higher. The mean UIR estimates for ISL in rats were A_1_ = 9.8 × 10^−4^ ng/mL/ng, A_2_ = 9.6 × 10^−6^ ng/mL/ng, α_1_ = 1.2 h^−1^ and α_2_ = 0.103 h^−1^.

### 3.3. In Vivo Absorption

**Figure 4:**
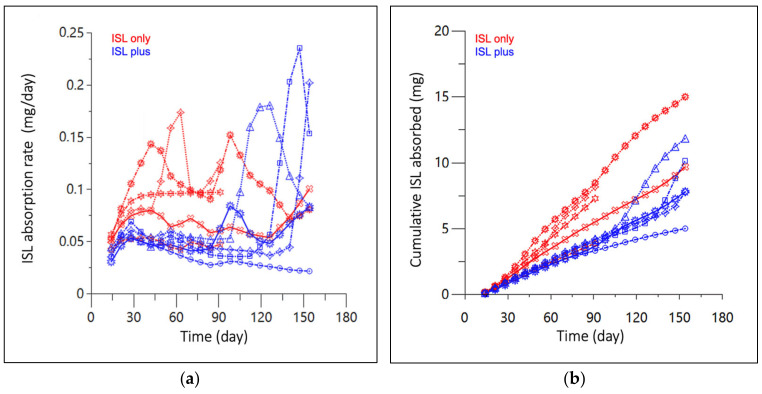
(**a**) Absorption rate, and (**b**) cumulative mass absorbed of subcutaneous ISL-eluting implants in rats over 154 days. Red represents animals receiving ISL only, while blue received ISL alongside a hormone implant (ISL plus). Lines and markers (shape) represent each individual animal sample collected at approximately 15-day increments. Three implants were removed after 100 days in the ISL only group.

The mean absorption rate from 0 to 154 days in the ISL-only and -plus groups were 0.084 ± 0.025 and 0.061 ± 0.018 mg/day with an overall mean of 0.072 ± 0.024 mg/day (Figure 4a). The mean cumulative mass absorbed from 0–154 days for the ISL-only and ISL-plus groups was 8.82 ± 4.07 and 8.53 ± 2.60 mg, respectively with an overall mean of 8.67 ± 3.22 mg (Figure 4b).

The best-fitting pseudo (linear) mathematical models describing in vivo absorption from the ISL-eluting implant were Zero-order and Ritger–Peppas (Table 1) with a mean (SD) r^2^ of 0.946 (0.04) and 0.985 (0.02), respectively. The mean (SD) power exponent estimated for Ritger–Peppas was 1.3 (0.29), with a non-reportable (N.R.) estimate for one animal due to non-convergence. First-order and Higuchi models did not meet the predetermined threshold of mean r^2^ > 0.75 with, mean r^2^ of −4.757 (1.08) and 0.699 (0.08), respectively. For the Higuchi model, only 3 of 10 animals had individual fits of r^2^ > 0.75. 

## 4. Discussion

While injectable LA PrEP, cabotegravir, helps address limitations to oral PrEP regimens, cost and bi-monthly clinical visits to administer the intramuscular injection by a trained professional may limit its utility among populations of lower socioeconomic status or living far from health infrastructures such as in rural areas [28]. The subtherapeutic PK tail of injectable LA PrEP is another disadvantage that poses a risk of breakthrough infection with ARV resistance in patients who switch therapy or fall out of care [8]. SC implants have been used clinically to deliver therapeutic hormonal contraception for up to 3 years since Nexplanon’s FDA approval in 2006 [29]. A similar delivery platform could improve upon injectable LA PrEP by extending the dosing interval, offering retrievability for those who choose to discontinue treatment, and facilitating multiple preventative indications such as contraceptive care. In clinical trials, an ISL-eluting implant was safe and achieved active metabolite (ISL-tp) concentrations in PBMCs above the PrEP efficacy threshold (0.05 pmol/million cells) for 12 weeks with projections that therapeutic exposure could be sustained for up to 16 months [17]. A downstream clinical trial of this implant recapitulated the clinical PK with no dose-dependent adverse events or discontinuations [18]. ISL remains a safe, potent and favorable model drug to develop as an implantable platform for LA HIV PrEP.

First-order release implants such as Implanon^®^ [9] are clinically useful; however, a zero-order implant would improve the predictability of drug release and may offer greater confidence in therapeutic concentrations near the end of the PK interval which may reduce risk of ARV resistance. Li et al. previously published on our reservoir-style, PCL biodegradable implant where zero-order release was directly observed for 13–17 months under in vitro sink conditions with demonstrable batch to batch consistency in raw material, formulation and end-product characteristics [22]. This release profile optimized implant delivered ISL for 6 months after subdermal implantation, was safe and biocompatible in a rat preclinical study [22]. While the original implant study was designed to explore whether dual implants had an influence on plasma PK, herein we sought to utilize a PK modeling approach with an additional independent SC bolus study to fully characterize in vivo absorption kinetics and guide optimization of drug release rates and dose translation efforts.

To estimate PK parameters of ISL in rats, we conducted a dose-ranging, SC bolus PK study of ISL solution that leveraged a sparse design with a population PK modeling approach. Single-dose pharmacokinetics indicated dose proportionality of ISL parent and its metabolite ISL-tp for all doses in rats [12]. ISL and ISL-tp halflife characterized in our study was limited by sample number and short duration of samples in the elimination phase. We observed a significant difference in rat PBMC ISL-tp: plasma ISL ratios compared to that reported in clinical trials of an ISL-eluting implant (~2390) [17]. However, our observation of lower ISL-tp:ISL ratios in rats relative to humans is consistent with the between species differences reported in Sykes et al. [24]. These findings indicate that allometric scaling of certain preclinical animal models will have limited utility in identifying clinical doses to achieve ISL’s PBMC efficacy target.

Based on the deconvolution model, only 3 of the 10 animals did not achieve an absorption rate of 50 μg/day for at least 50% of the analyzed profile. Graphical analysis of Figure 7c,d in Barrett et al. demonstrated that a SC ISL-eluting implant releasing approximate 250 μg/day was projected to achieve 2 nM ISL plasma concentrations in humans 60 days post-implantation [30]. During first in human trials of their down-selected implant formulation, steady-state ISL plasma concentrations of 1 nM were associated with ISL-tp PBMC concentrations of 0.272 pmol/million cells, which is 5-fold higher than target exposure [17]. Given our average dose of 50 μg/day, and assuming no between-species differences in drug delivery mechanisms, we predict that our implant will result in human PBMC concentrations at or exceeding the PrEP threshold of 0.05 pmol/million cells. Furthermore, all estimated absorption rates throughout the 154 day observation window were below 0.20 mg/day, with the exception of one timepoint that approached, but was still below the 0.25 mg/day upper dosing limit for oral ISL. In examining the PK curve associated with this observation it is likely that impaired mechanical integrity of the implant contributed to this isolated observation of abnormally high release [22]. Cumulative drug release estimated by deconvolution in our study showed that less than 15% of the mean loaded dose (98.6 mg) was absorbed during the 154 day observation window. Consistent with that estimation, analysis of residual ISL in recovered implants from three animals at a 3-month interim necropsy demonstrated 3–7% (min–max) of ISL had been absorbed. Fine-tuning an ARV-eluting implant to deliver a constant rate of the lowest effective dose within an in vivo system is a worthwhile developmental goal to conserve ARV payload and extend the dosing interval. Herein, we provide in vivo evidence that a reservoir-style implant comprised of PCL can deliver ISL at a constant rate that falls within the safe and potentially effective dosing range for humans for up to 5 months. Furthermore, this implant can be loaded with sufficient ISL to sustain this release profile for well over a year.

ISL’s in vivo absorption was consistent with a constant rate drug release as the zero-order equation explained a high proportion of variance in our data with r^2^ values ranging from 0.87–0.99. Because biofluids transport into our implant architecture via passive diffusion, swelling is possible. Thus, we also tested a Ritger–Peppas model of mass transport intended to deal with the moving boundaries problem that swelling presents for Fickian diffusion. Importantly, the estimated power exponent term within our Ritger–Peppas model fits approximated 1 with a mean (SD) of 1.3 (0.29). This finding further supports our conclusion of time-independent constant rate transport (i.e., zero-order) [31]. For cylindric drug delivery systems such as ours, Ritger–Peppas exponential terms of 2 are associated with the time-dependent Super Case-II release, terms of 0.45–0.89 with non-Fickian anomalous release and terms of 1 with zero-order release [31]. Furthermore, the Higuchi equation explained a relatively low proportion of variance in our data with r^2^ values ranging from 0.59 to 0.83. Finally, within our first-order model fits, a constant function better fit the data than a linear one leading to negative r^2^ values. Ultimately, the overall zero-order controlled release mechanism characterized by our PCL, biodegradable, reservoir-style implant may improve the predictability of estimating PK beyond the observation period, assuming polymer and physiologic conditions are optimized. While implant constant rate kinetics may become less predictable when physiochemical conditions change, i.e., the implant polymer degrades, and/or the payload decreases, the current polymer has been adjusted for persistent physiological conditions up to 1 year. Historically, reservoir-style architecture provides the greatest predictability of drug PK for implantable polymeric systems [26]. Our zero-order controlled release implant has leveraged this advantage to improve prediction of in vivo ISL absorption and guide drug development of a LA-implantable PrEP device.

While unquantifiable ISL concentrations at 24 h following the 0.1 mg/kg SC dose increased residual model error, downstream analyses were determined by population estimates that met assumptions of linearity and normality per the diagnostics plots (Figure 3b–d). Differences in T_max_, 10 min vs. 30 min, are likely attributed to the study size and random variability due to sparse sampling methods. While an individual UIR estimation for each rat used in the implant study would be an ideal study design, blood volume constraints precluded the intensive sampling necessary to accurately estimate ISL’s UIR in individual animals. The Wistar rats utilized in the two separate studies were sourced from a reputable vendor and the studies were conducted within the same animal facility using identical sample collection processes. There was <20% difference in the range of animal weights in the subcutaneous bolus and implant studies [22]. Deconvolution profiles (Figure 4a,b) elucidated between group differences for mean absorption rate (*p* = 0.03) and cumulative mass absorbed at 90 days (*p* = 0.03), that were not statistically different at 154 days, *p* = 0.12 and *p* = 0.89, respectively. Since implant PCL fabrication, ISL formulation and animal cohorts were matched across both animal groups and interrogated across several in vitro studies, no biopharmaceutical reasons have been identified for these differences and are likely attributed to mismatched numbers in groups [22]. The parallel in vitro ISL dissolution study conducted by Li et al. reported a mean in vitro release rate of 0.066 ± 0.008 mg/day, which compares well to our deconvolved mean in vivo absorption rate of 0.072 ± 0.024 μg/day indicating a high correlation of drug release kinetics from in vitro-to-in vivo [22]. This PK modeling approach identified a direct relationship between in vitro and in vivo absorption rates.

The predictive applicability of deconvolution is associated with estimating a suitable UIR. Because our UIR is derived from subcutaneous PK data, our model relies on an assumption of near instantaneous absorption. Our estimated absorption (Ka) value of 12 hr^−1^ provides evidence of instantaneous absorption of ISL into the plasma from the subcutaneous space. While sampling was limited in the SC bolus rat study, the observed data was developed into a fit-for-purpose model describing the mean population parameters necessary for deconvolution. Additionally, dose level half-life estimation was limited by sparse sampling in the elimination phase and was not utilized as a PK parameter for deconvolution. The sparse observed data prior to C_max_ indicated rapid absorption of ISL 10–30 min after SC bolus administration for all doses. Future studies present an opportunity to intensely sample the absorption and terminal phases to improve robustness for applications that require a full PK model. Though limited literature is available on the individual acceptability of UIRs estimated from extravascular routes of administration, various extended release models have been reported that leverage extravascular routes of administration for deconvolution purposes [32,33]. Additionally, European and U.S. agencies accept UIR derived from extravascular routes of administration for regulatory review purposes [34,35]. The applicability of our model to inform dose-translation efforts also relies on an assumption that SC absorption rates do not differ between species. This approach to derive in vivo absorption has been successful in translating dosing from preclinical to clinical phases [17,30]. Altogether, we found the deconvolution methods conducted herein to be highly applicable to this platform and do not expect dose translation to be inhibited by use of a SC dose-ranging design for PK analysis and UIR determination.

Novel LA PrEP platforms with simplified dosing are critical for addressing adherence challenges in HIV prevention. The PK modeling approach applied herein demonstrates that our reservoir-style biodegradable ISL-eluting implant can sustain zero-order release at least 30 μg/day for 5 months in rats. We project that this implant could achieve clinical exposure exceeding ISL’s PrEP efficacy threshold. Li et al. previously demonstrated the utility of this implant design for sustained zero-order hormonal contraceptive delivery [22]. This deconvolution approach estimated the daily dose of a prototype implant designed to deliver extended-duration antivirals. Together, these data support the utility of PK modeling as a guide to preclinical development of this implant device as a promising candidate for co-formulation and downstream applications in multipurpose prevention technologies (MPT) including LA HIV PrEP.

## Figures and Tables

**Table 1:** Regression coefficient (r^2^) kinetic models of drug absorption from the SC ISL eluting implant.

Group	Zero-Order r^2^	First-Order r^2^	Higuchi r^2^	Ritger–Peppas ^b^ r^2^
ISL_only	0.9680	−6.6068	0.7054	N.R. ^a^
ISL_only	0.8933	−4.0118	0.5972	0.9909
ISL_only	0.9409	−4.5061	0.6568	**0.9944**
ISL_only	0.9776	−3.0757	0.7388	0.9908
ISL_only	**0.9888**	−4.3850	0.7672	0.9942
ISL_plus	0.9805	−6.5246	0.8384	0.9855
ISL_plus	0.8667	−4.2326	0.5859	0.9844
ISL_plus	0.8971	−4.7319	0.6481	0.9399
ISL_plus	**0.9825**	−4.8870	0.7619	0.9878
ISL_plus	0.9615	−4.6075	0.6997	**0.9978**
Mean	**0.9457**	−4.7569	0.6999	**0.9851**
Standard deviation	0.04	1.08	0.08	0.02

Best results in bold. ^a^ N.R. not-reportable due to model non-convergence. ^b^ Mean (SD) power exponent, *n*, of 1.3 ± 0.29.

## Data Availability

The data presented in this study are openly available online in the National Library of Medicine, PubMed at [https://doi.org/10.1016/j.jconrel.2021.10.021, accessed on 12 October 2023], reference number [22].

## Supplementary Materials

The following supporting information can be downloaded at: https://www.mdpi.com/article/10.3390/pharmaceutics16020201/s1, Figure S1: Model code for regression analysis of 4 kinetic models of drug distribution in SAS^TM^ 9.4.; Table S1: Population pharmacokinetic (PopPK) model development and comparison in Phoenix NLME^TM^.
