# Supplementary material for: Pharmacokinetic Modeling to Guide Preclinical Development of an Islatravir-Eluting Reservoir-Style Biodegradable Implant for Long-Acting HIV PrEP
Source: Pharmaceutics. 2024 Jan 30;16(2):201. doi: 10.3390/pharmaceutics16020201 (PMC10893066; doi:10.3390/pharmaceutics16020201)
Supplement: Supplementary file 1 [file pharmaceutics-16-00201-s001.zip › pharmaceutics-2607283-supplementary.pdf]

*Pharmaceutics*
Supplementary Material

| No. | | Model | Param | -2LL | AIC | BIC | Diagnostics | | | | RSE |
|---|---|---|---|---|---|---|---|---|---|---|---|
| 1 | **Independent Dose Models** | 0.1 mg/kg<br>2CMT NPD<br>Multi | 6 | 70.7 | 82.7 | 88.1 | 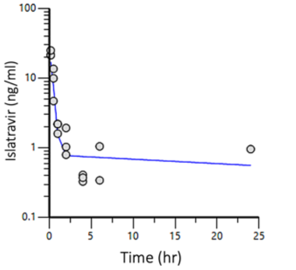 | 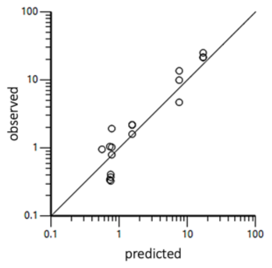 | 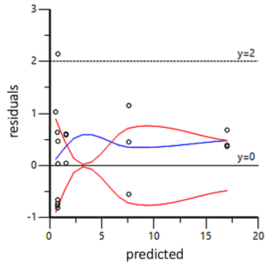 | 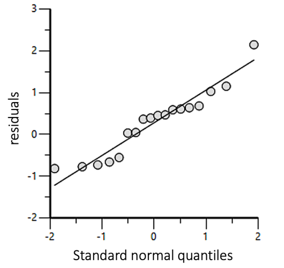 | 0.69 |
| 2 | | 0.2 mg/kg<br>2CMT NPD<br>Multi | 6 | 81.1 | 93.8 | 98.8 | 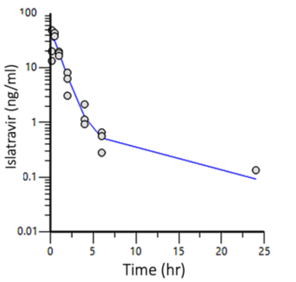 | 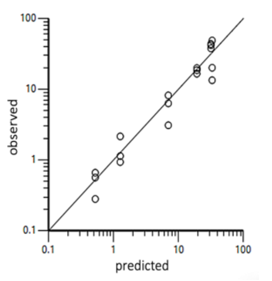 | 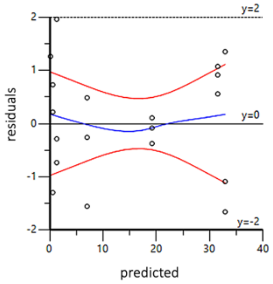 | 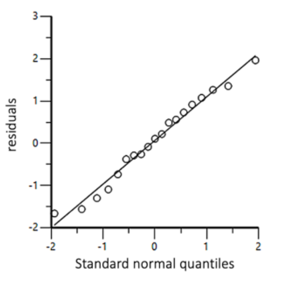 | 0.36 |
| 3 | | 1.0 mg/kg<br>2CMT NPD<br>Multi | 6 | 94.1 | 106.1 | 112.4 | 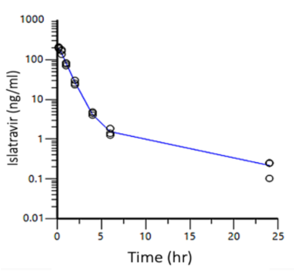 | 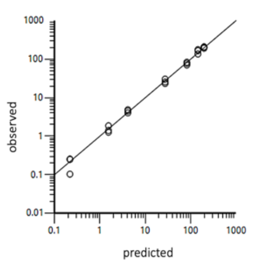 | 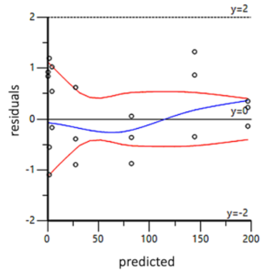 | 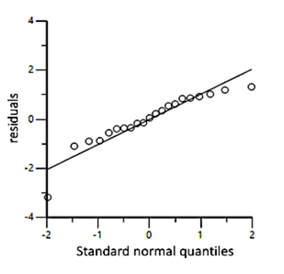 | 0.17 |

| No. | | Model | Param | -2LL | AIC | BIC | Diagnostics | RSE |
|---|---|---|---|---|---|---|---|---|
| 4 | Simultaneous Dose Model Development | 1CMT NPD Multi | 4 | 559.1 | 567.1 | 575.9 | 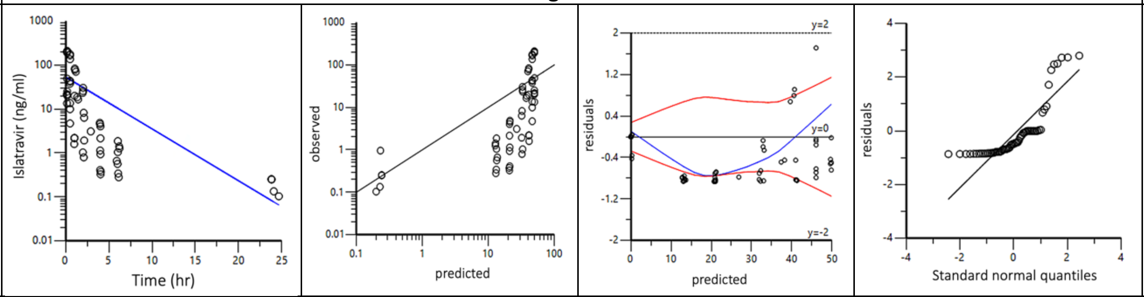 | 1.14 |
| 5 | | 2CMT NPD Multi [Base Model] | 6 | 414.8 | 426.4 | 438.7 | 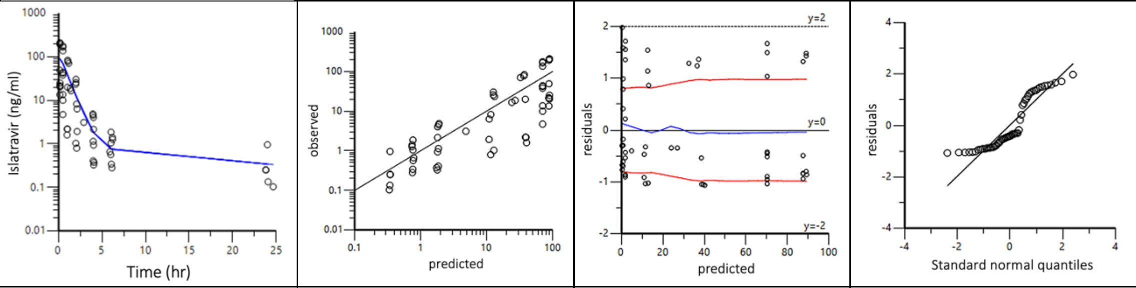 | 0.90 |
| 6 | Final Model | Base Model no Outlier | 6 | 423.7 | 435.7 | 448.9 | 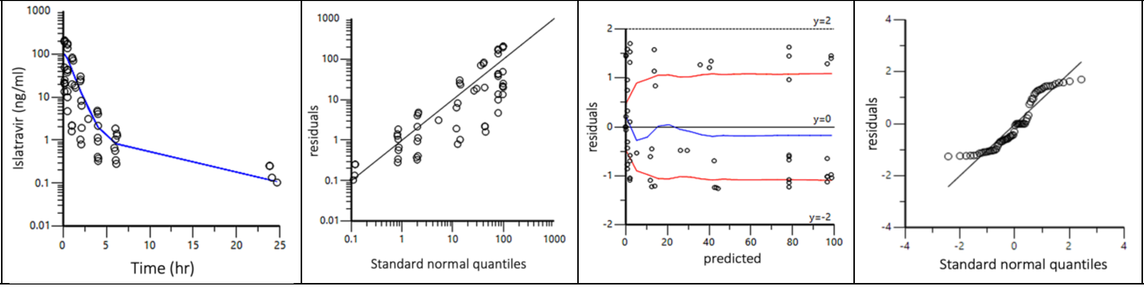 | 0.76 |

**Base Model vs. Final Model Theta Parameters**

| | Final | Base | Final | Base | Final | Base | Final | Base | Final | Base |
|---|---|---|---|---|---|---|---|---|---|---|
| | Ka (1/h) | | A (ng/ml) | | alpha (1/h) | | B (ng/ml) | | beta (1/h) | |
| **Estimate** | 12.23 | 12.79 | 141.60 | 125.66 | 1.21 | 1.18 | 1.39 | 0.83 | 0.103 | 0.037 |
| **Std error** | 2.06 | 2.12 | 36.34 | 35.83 | 0.06 | 0.05 | 0.33 | 0.30 | 0.007 | 0.023 |
| **CV%** | 16.83 | 16.59 | 25.7 | 28.5 | 4.6 | 4.1 | 24.1 | 36.4 | 6.6 | 64.0 |
| **2.5% CI** | 8.11 | 8.53 | 68.92 | 53.76 | 1.10 | 1.08 | 0.72 | 0.22 | 0.089 | -0.010 |
| **97.5% CI** | 16.34 | 17.05 | 214.28 | 197.57 | 1.32 | 1.28 | 2.06 | 1.43 | 0.117 | 0.083 |

*Circles are observed data; blue lines are predicted curves; red lines are distribution of residuals. CMT- compartment; NPD- naïve pooled; RSE- relative standard error; Ka- absorption rate constant; A- 1st CMT macroconstant; B- 2nd CMT macro-constant; alpha/beta- elimination rate constant; CI- confidence interval.*

**Table 1: Population pharmacokinetic (PopPK) model development and comparison in Phoenix NLME™.**

The population pharmacokinetic (PopPK) analyses were performed using Phoenix NLME modeling software. The model building process was a stepwise approach: independent modeling of islatravir plasma concentration for the three subcutaneous bolus doses (0.1, 0.3 and 1mg/kg), identification of the most appropriate structural base model followed by developing a simultaneous base model of the collapsed dose range. The base structural model was tested for 1, 2 and 3 compartments, describing first-order absorption and linear elimination. The structural model was developed with a fit-for-purpose approach to deconvolution leveraging macro-parameterization to estimate; first-order absorption rate constant (Ka), macro-constants (A and B), elimination rate constants (alpha and beta). A 2-compartment structural models provided a better fit for the collapsed simultaneous model of 0.1, 0.3 and 1.0mg/kg dosing levels. Residual diagnostic plots of the 2-comp Final model show some bias in the fit but do not indicate misspecification as with the 1-comp comparator. A 3 Finally, while AIC increases with addition of a second compartment the-2Loglikelihood decreases (-113). Overall, the fits and criteria indicate an improvement with a 2-compartment structure. Final overall model error decreases from 113% to 76% from a 1 to 2-compartment model when no other factors were changed.

Zero order

```
proc model data=work.RatKinetic;
 by Ind;
 parms K0=42576;
 Mt= K0*t;
 fit Mt;

 run;
```

First order

```
proc model data=work.RatKinetic;
 by Ind;
 parms Kf=.01;
 LnMr=Kf*t;
 fit LnMr;
 run;
```

Higuchi

```
proc model data=work.RatKinetic;
 by Ind;
 parms Kh=1000;
 Mt=Kh*(t**.5);
 fit Mt;
 run;
```

Ritgers-Peppas

```
proc model data=work.RatKinetic;
 by Ind;
 parms Krp=0.001 n=0.45;
 Ft = Krp*(t**n);
 fit Ft;
 run;
```

**Figure 1: Model code for regression analysis of 4 kinetic models of drug distribution in SAS™ 9.4.**
Top down shows each model formula and initial estimate used to fit a regression using the ordinary least squared function. Where *Mt* is the rate constant of each respective model, *Ft* is the ratio of *Mt/Minf* (the mass loaded in the implant), *n* is the power exponent of release, and
